# Intraoperative ultrasonography in the surgical management of Chiari I malformation: A systematic review and meta-analysis of outcomes and applications

**DOI:** 10.1007/s10072-026-08879-8

**Published:** 2026-03-09

**Authors:** Bardia Hajikarimloo, Ibrahim Mohammadzadeh, Mohammad Amin Habibi, Salem M. Tos, Haris Yaseen, Nathan Chisvo, Mohammadmahdi Sabahi, Qais Alrashidi, Badih Adada, Hamid Borghei-Razavi

**Affiliations:** 1 Department of Neurological Surgery, University of Virginia, Charlottesville, VA USA; 2 Skull Base Research Center, Loghman-Hakim Hospital, Shahid Beheshti University of Medical Sciences, Tehran, Iran; 3 Department of Neurosurgery, Shariati Hospital, Tehran University of Medical Sciences, Tehran, Iran; 4 Department of Neurosurgery, Mayo Hospital, Lahore, Pakistan; 5 Barts and the London School of Medicine and Dentistry, Queen Mary University of London, London, UK; 6 Department of Neurological Surgery, Pauline Braathen Neurological Center, Cleveland Clinic Florida, Weston, FL USA; 7 Cleveland Clinic Lerner College of Medicine of Case Western Reserve University, Cleveland, OH USA; 8 Neurological Surgery, CCLCM of CWRU Director of Minimally Invasive Cranial and Pituitary Surgery Program Research Director, Neuroscience Institute, Cleveland Clinic Florida Region, Cleveland, OH USA

**Keywords:** Chiari malformation type I, Intraoperative ultrasonography, Syringomyelia, Meta-Analysis

## Abstract

**Background:**

Chiari malformation type I (CM-I) is a neurological disorder characterized by cerebellar tonsillar herniation and is often associated with syringomyelia. Posterior fossa decompression (PFD) is the primary treatment, although the optimal extent of decompression remains a topic of debate. Intraoperative ultrasonography (IOUS) offers real-time visualization to support informed surgical decision-making. This study aimed to assess the effectiveness and safety of IOUS-guided surgery in CM-I.

**Methods:**

This systematic review followed PRISMA guidelines and searched the literature up to July 5, 2025. Studies that utilized IOUS and reported related outcomes were included. A random-effects meta-analysis was employed to pool proportions, and a meta-regression analysis was conducted to explore heterogeneity.

**Results:**

Twenty-one non-randomized observational studies, including 1,576 patients, 54.4% of whom were female, were included. The pooled clinical improvement rate was 88% (95% CI: 81%–93%), and the syrinx improvement/resolution rate was 87% (95% CI: 77%–95%). The pooled reoperation rate was 6% (95% CI: 3%–9%), and the complication rate was 6% (95% CI: 3%–8%). Meta-regression revealed that longer disease duration, motor/sensory deficits, and duraplasty were associated with improved syrinx outcomes.

**Conclusion:**

IOUS-guided surgery in CM-I is linked to positive clinical and radiological improvements and low complication rates. IOUS can serve as a helpful tool for customizing decompression during CM-I surgery. Larger, prospective, multi-center studies are needed to confirm these findings.

**Graphical Abstract:**

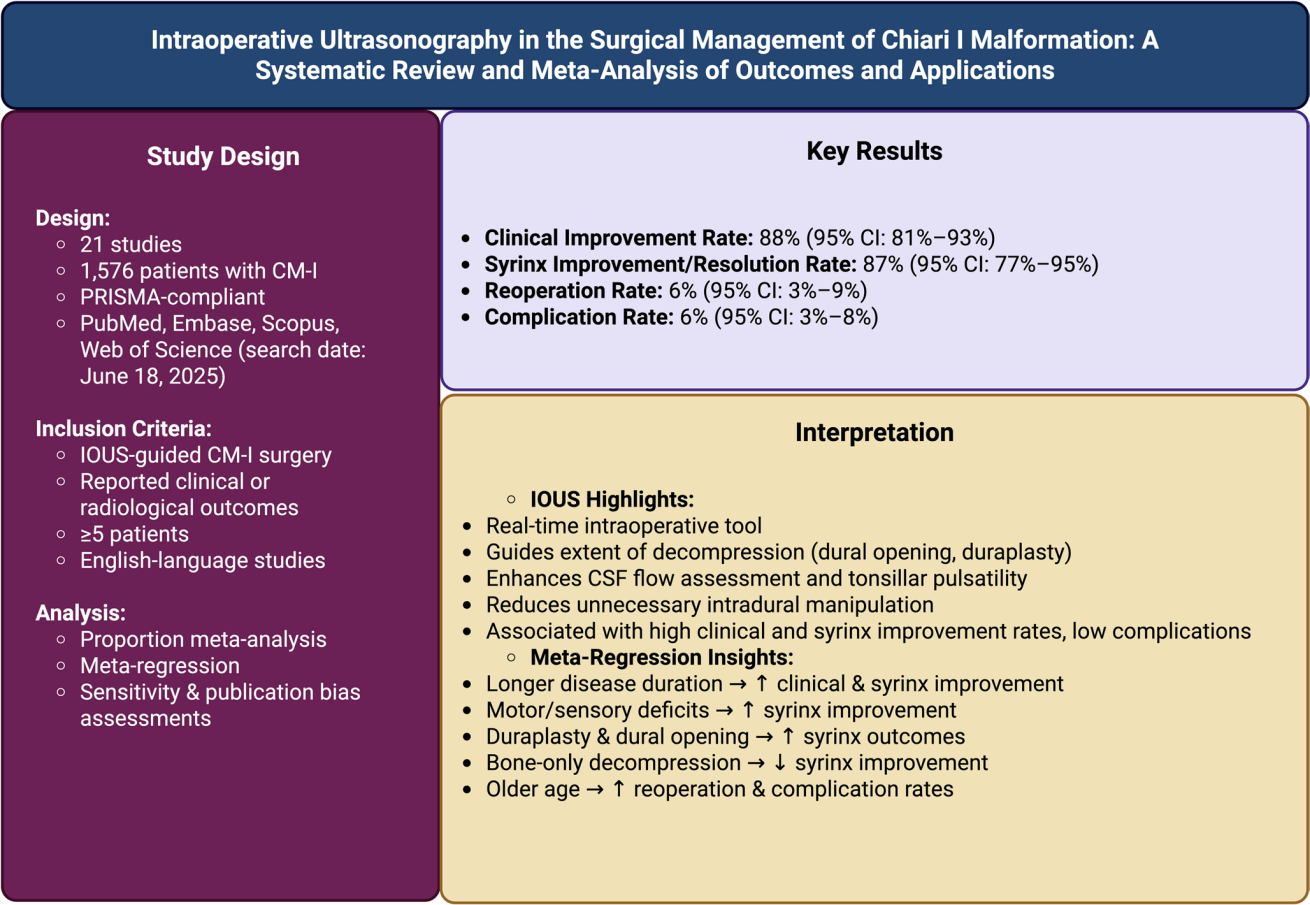

**Supplementary Information:**

The online version contains supplementary material available at 10.1007/s10072-026-08879-8.

## Introduction

Chiari malformation type I (CM-I) is a congenital structural disorder of the hindbrain, characterized by herniation of the cerebellar tonsils ≥ 5 mm below the foramen magnum [1–3]. CM-I is associated with a prevalence rate of approximately 0.5% to 3.5% of the general population, and it is increasing due to advancements and higher utilization of magnetic resonance imaging (MRI) [4, 5]. Many CM-I patients are asymptomatic; however, others present with symptoms such as headache, neck pain, cerebellar dysfunction, or sensory and motor deficits [6–9]. Syringomyelia, which is a fluid-filled cavity in the spinal cord, is observed in 50–80% of symptomatic CM-I patients and is usually an indication for surgical intervention [2, 9, 10].

According to the recent international consensus by Ciaramitaro et al., the main clinical and radiological subtypes include CM-I, a paraxial mesodermal disorder characterized by a small posterior fossa and cerebellar tonsillar descent of ≥ 5 mm below the foramen magnum, subdivided into CM-I A (with syringomyelia) and CM-I B (without syringomyelia); CM-II, associated with spinal dysraphism and prenatal cerebrospinal fluid hypotension; Chiari 1.5 or complex Chiari, defined by tonsillar descent with brainstem kinking and craniovertebral junction anomalies such as basilar invagination or atlanto-occipital fusion; and Chiari 0, characterized by syringomyelia without tonsillar herniation but with posterior fossa crowding or arachnoid adhesions [11]. Additionally, acquired tonsillar ectopia, also known as acquired Chiari, refers to the downward displacement of the tonsils secondary to intracranial space-occupying lesions or hydrocephalus [11]. Radiologically, CM-I is diagnosed on midsagittal MRI as ≥ 5 mm tonsillar descent below the basion-opisthion line, while 3–5 mm indicates borderline ectopia, especially when accompanied by a syrinx or peg-like tonsils [11]. Clinically, the term “Chiari syndrome” refers to symptomatic CM-I, which presents with Valsalva-induced occipital headaches, cerebellar or brainstem dysfunction, and spinal manifestations often associated with syringomyelia, reflecting the integrated clinicoradiologic framework emphasized by the international consensus [11].

The primary therapeutic option for CM-I patients is posterior fossa decompression (PFD) with or without duraplasty [12–14]. However, the extent of decompression, particularly whether to open the dura and perform duraplasty, remains controversial because of the risk of cerebrospinal fluid (CSF) leakage, pseudomeningocele, and infection. Intraoperative Ultrasonography (IOUS) has been used as a valuable tool during surgery to improve decision-making by providing real-time visualization of CSF flow and cerebellar tonsillar pulsations [15–35]. The application of IOUS enables surgeons to personalize the decompression extent that can mitigate the risk of adverse events while maintaining favorable clinical results.

Despite the increasing use of IOUS in surgical interventions for CM-I patients, its role remains inadequately defined. Variations in practice protocols, surgical approaches, and reporting standards across studies have contributed to uncertainty about its overall effectiveness. This systematic review and meta-analysis assess the existing evidence regarding the use of IOUS in CM-I surgery by synthesizing data on clinical outcomes, Syringomyelia outcomes, reoperations, and complications. By analyzing pooled estimates, this study aims to provide clearer guidance on the value of IOUS in current CM-I surgical practice.

## Materials and methods

### Objective

Following the “Preferred Reporting Items for Systematic Reviews and Meta-Analyses (PRISMA)” guidelines, we evaluated the role of IOUS in CM-I surgery [36].

### Search strategy

On July 5, 2025, a thorough literature search was performed in PubMed, Embase, Scopus, and Web of Science. The search included “Arnold-Chiari Malformation,” “Intraoperative Period,” “Ultrasonography,” and their equivalents. The Supplementary Table S1 contains the search queries for each database.

### Eligibility criteria

The PICO framework is provided in Supplementary Table S2. Inclusion criteria included studies involving at least five patients with CM-I undergoing surgical decompression that explicitly mention the use of IOUS in the manuscript text, published in 1990 or later, and reporting at least one of the following outcomes: clinical improvement, syrinx resolution, reoperation, or complication rates. Exclusion criteria included reviews, book chapters, conference abstracts, preprints, and editorials; non-human or cadaveric studies; studies lacking extractable outcome data related to IOUS in CM-I surgery; studies with overlapping participant populations; and publications not in English. To identify overlapping populations, we examined study institutions, author groups, recruitment periods, and reported patient characteristics; when overlap was detected, only the most comprehensive or most recent dataset was included.

### Study selection process, data extraction, and risk of bias assessment

After searching the literature, the results were imported into the Covidence software. Covidence identified and removed duplicates. Next, two independent reviewers conducted the screening of titles and abstracts, excluding studies that did not meet the inclusion criteria. Following this, the remaining studies were evaluated through full-text assessment, and those that satisfied the inclusion criteria were included for data extraction. The variables are listed in Supplementary Table S3. The definitions of the outcomes are provided in the Supplementary Table S4. The Risk of Bias (RoB) assessment for the selected studies was performed using the Methodological Index for Non-Randomized Studies (MINORS) tool [37].

### Statistical analysis

The R (version 4.4.2) was used for the study analysis with the “meta” and “metafor” packages. Study proportions were pooled using inverse-variance weighting with the Freeman–Tukey double arcsine transformation (metaprop, sm="PFT”); random-effects CIs used the Hartung–Knapp adjustment. This approach was chosen to stabilize variance and handle boundary proportions. A proportion meta-analysis was performed to obtain a pooled estimate for each endpoint, along with 95% confidence intervals (CIs). Heterogeneity was assessed using the I² statistic and Cochran’s Q test, with I² values over 40% or P less than 0.1 indicating significant heterogeneity. Leave-one-out sensitivity analysis was conducted to test the robustness of the pooled estimates. Publication bias was checked with funnel plots and Egger’s test. Meta-regression was utilized to identify potential sources of heterogeneity, with significant moderators defined by p-values less than 0.05. To standardize parameters, if a variable was reported as a median, it was converted to a mean using the method of Luo et al. [38]. A Grading of Recommendations, Assessment, Development and Evaluations (GRADE) assessment was performed to evaluate the overall certainty of evidence for each pooled outcome.

## Results

### Study selection process

**Fig. 1:**
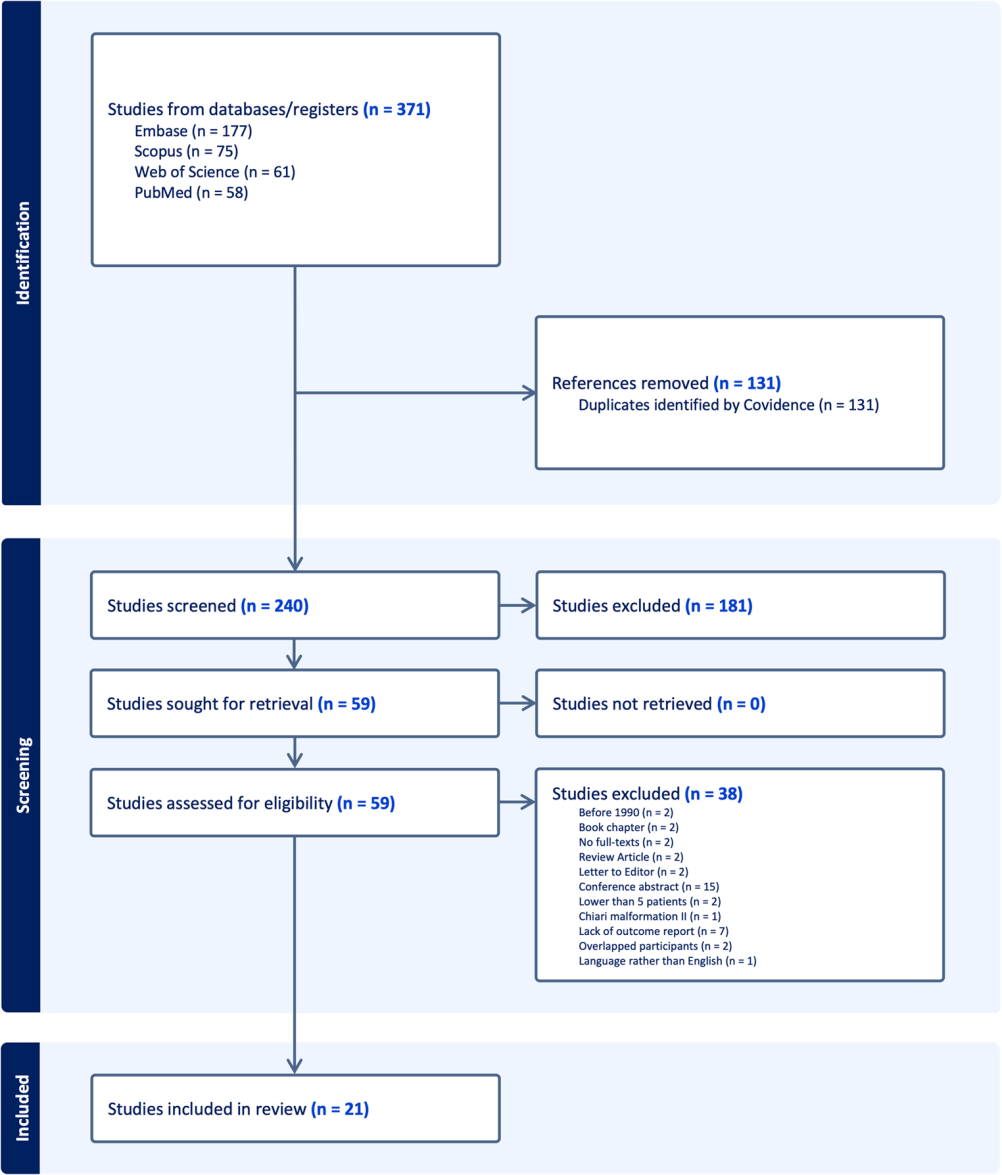
PRISMA flowchart of the included studies

The search yielded 371 studies from four electronic databases (Fig. 1). Of these, 131 were identified as duplicates and were excluded accordingly. Thus, 240 studies were included for title and abstract screening. During this step, 181 did not meet the inclusion criteria and were excluded, and 59 studies were included for full-text screening. During full-text screening, 38 were excluded, of which 15 were abstracts, two were published before 1990 [39, 40], two were for consisting of fewer than five patients [41, 42], and two overlapped in participants [43, 44]. Ultimately, 21 studies met the inclusion criteria and were included [15–35].

### Risk of bias assessment

The RoB assessment indicated that most of the included studies had moderate RoB (Supplementary Table S5). The RoB assessment revealed that the overall scores ranged from 14/24 to 22/24. Seven studies were rated as high quality, nine as moderate quality, and four as low quality. Common methodological limitations included a lack of prospective calculation of study size, absence of blinded outcome assessment, and incomplete follow-up reporting. These findings suggest that, although some studies demonstrated strong methodological rigor, variability in reporting and potential biases should be taken into account when analyzing the pooled results.

### Baseline characteristics

Twenty-one studies with 1,576 patients were included (Table 1). In the included studies, 66.7% (14/21) were retrospective and 33.3% (7/14) were prospective studies. The United States (52.4%, 11/21) was the most frequent country of origin of the included studies. The mean age ranged from 5.9 to 48.2 years in the included cohorts. The female gender comprised 54.4% (674/1239) of the included cohort. The mean time from the onset of symptoms to surgery ranged from 0.4 to 7.1 years. Headache was present in 64.9% (770/1187), motor deficit was observed in 29.9% (175/585), sensory deficit was in 42.7% (244/571), and cerebellar dysfunction was seen in 19.4% (155/801) of the cases. The syrinx was observed in 50.1% (789/1576) of the cases. Hydrocephalus was present in 7.2% (89/1239) of the cases.

**Table 1:** Baseline characteristics of included studies on IOUS in CM-I

Study	Year	Country	Design	Mean Age	Patients (M/F)	Mean Disease Duration (mo)	Headache	Motor Deficit	Sensory Deficit	Cerebellar Signs	Cranial Nerve Symptoms	Pre-op Syrinx	Pre-op Hydrocephalus	Bone-Only Decompression	Duraplasty	Dural Opening
Venanzi et al.	2024	Italy	R	8.1	211 (122/89)	NA	145	NA	NA	61	NA	60	17	119	74	80
Jha et al.	2024	USA	R	NA	15 (1/14)	NA	11	3	6	3	3	9	NA	7	8	8
Dherijha et al.	2024	UK	R	47	57 (9/48)	3.37	54	NA	22	10	8	21	0	52	3	3
Szuflita et al.	2021	USA	R	NA	115 (NA/NA)	NA	NA	NA	NA	NA	NA	115	NA	40	43	75
Liu et al.	2020	China	R	40.6	78 (36/42)	NA	45	30	32	11	8	78	15	0	78	78
Salomão et al.	2019	Brazil	R	8.6	24 (14/10)	NA	16	14	NA	NA	NA	15	3	18	6	6
Knerlich-Lukoschus et al.	2019	Germany	R	13	22 (11/11)	NA	10	NA	6	2	1	11	0	22	0	0
Dlouhy et al.	2018	USA	P	17.6	123 (42/81)	NA	NA	NA	NA	NA	NA	89	0	0	123	123
Brock et al.	2017	Brazil	P	48.2	49 (19/30)	1.2	29	31	30	16	11	22	NA	36	13	13
Fan et al.	2017	China	R	38	126 (62/64)	NA	68	32	66	6	11	90	NA	0	NA	78
Barzilai et al.	2016	Israel	R	NA	21 (8/13)	NA	NA	NA	NA	NA	NA	18	0	0	21	22
Kennedy et al.	2015	USA	R	9.9	156 (80/76)	2.3	65	19	38	23	40	68	7	156	0	0
Narenthiran et al.	2015	UK	R	10.5	19 (10/9)	NA	8	2	NA	2	2	14	0	11	0	8
Parker et al.	2013	USA	P	38.5	50 (14/36)	NA	50	20	NA	NA	13	20	3	6	44	44
Cui et al.	2011	China	P	44.65	20 (11/9)	7.1	8	9	14	7	3	18	NA	1	19	19
Heiss et al.	2010	USA	P	35	16 (NA/NA)	NA	10	5	14	6	NA	16	0	3	13	16
McGirt et al.	2008	USA	R	10	256 (121/135)	1	192	NA	NA	NA	68	69	35	16	140	140
Yeh et al.	2006	USA	R	5.9	130 (NA/NA)	NA	NA	NA	NA	NA	NA	27	9	40	85	86
Limonadi et al.	2004	USA	P	7.6	12 (5/7)	NA	11	4	3	6	2	0	NA	0	0	12
Navarro et al.	2004	USA	R	NA	56 (NA/NA)	NA	40	NA	NA	NA	NA	9	0	0	0	0
Heiss et al.	1999	USA	P	34.6	20 (NA/NA)	3.2	8	6	13	2	NA	20	0	0	20	20

NA: Not available; P: Prospective; R: Retrospective; M/F: Male/Female; mo: months; Pre-op: Preoperative

### Clinical and radiological outcomes

The mean follow-up period ranged from 6.45 to 70.8 months in the included studies (Table 2). The clinical improvement rate varied from 66% to 100%, and the syrinx improvement rate ranged from 45.5% to 100% in the cohort. The reoperation rate varied from 0.0% to 28.6%, and the complication rate ranged from 0.0% to 18.0% across the included studies.

**Table 2:** Outcome characteristics of included studies on IOUS in CM-I

Study	Mean FU (months)	Clinical Improvement (%)	Syrinx Improvement/Resolution (%)	Reoperation (%)	Complication (%)
Venanzi et al.	50.40	92.9%	91.7%	2.8%	3.3%
Jha et al.	36.00	92.9%	100%	0%	0%
Dherijha et al.	38.40	98.2%	76.2%	12.3%	10.5%
Szuflita et al.	NA	93.9%	NA	NA	NA
Liu et al.	22.80	84.6%	87.2%	28.6%	15.4%
Salomão et al.	70.80	79.2%	NA	8.3%	16.7%
Knerlich-Lukoschus et al.	NA	NA	45.5%	4.5%	0%
Dlouhy et al.	57.60	NA	NA	1.6%	2.4%
Brock et al.	12.00	72.5%	NA	15%	7.5%
Fan et al.	24.80	NA	86.7%	4.8%	9.5%
Barzilai et al.	NA	NA	NA	4.5%	4.5%
Kennedy et al.	32.00	90.8%	70.2%	9.2%	2.6%
Narenthiran et al.	12.00	69.2%	71.4%	10.5%	5.3%
Parker et al.	12.00	66%	NA	18%	18%
Cui et al.	12.00	95%	100%	0%	0%
Heiss et al.	6.45	93.8%	93.8%	6.2%	6.2%
McGirt et al.	29.00	78.1%	NA	7.4%	3.1%
Yeh et al.	20.00	95.2%	80.8%	4.6%	9.2%
Limonadi et al.	15.70	NA	NA	0%	0%
Navarro et al.	27.60	71.4%	NA	14.3%	7.1%
Heiss et al.	39.60	100%	100%	0%	10%

FU: Follow-Up; NA: Not Available

### Meta-analysis of outcomes

**Fig. 2:**
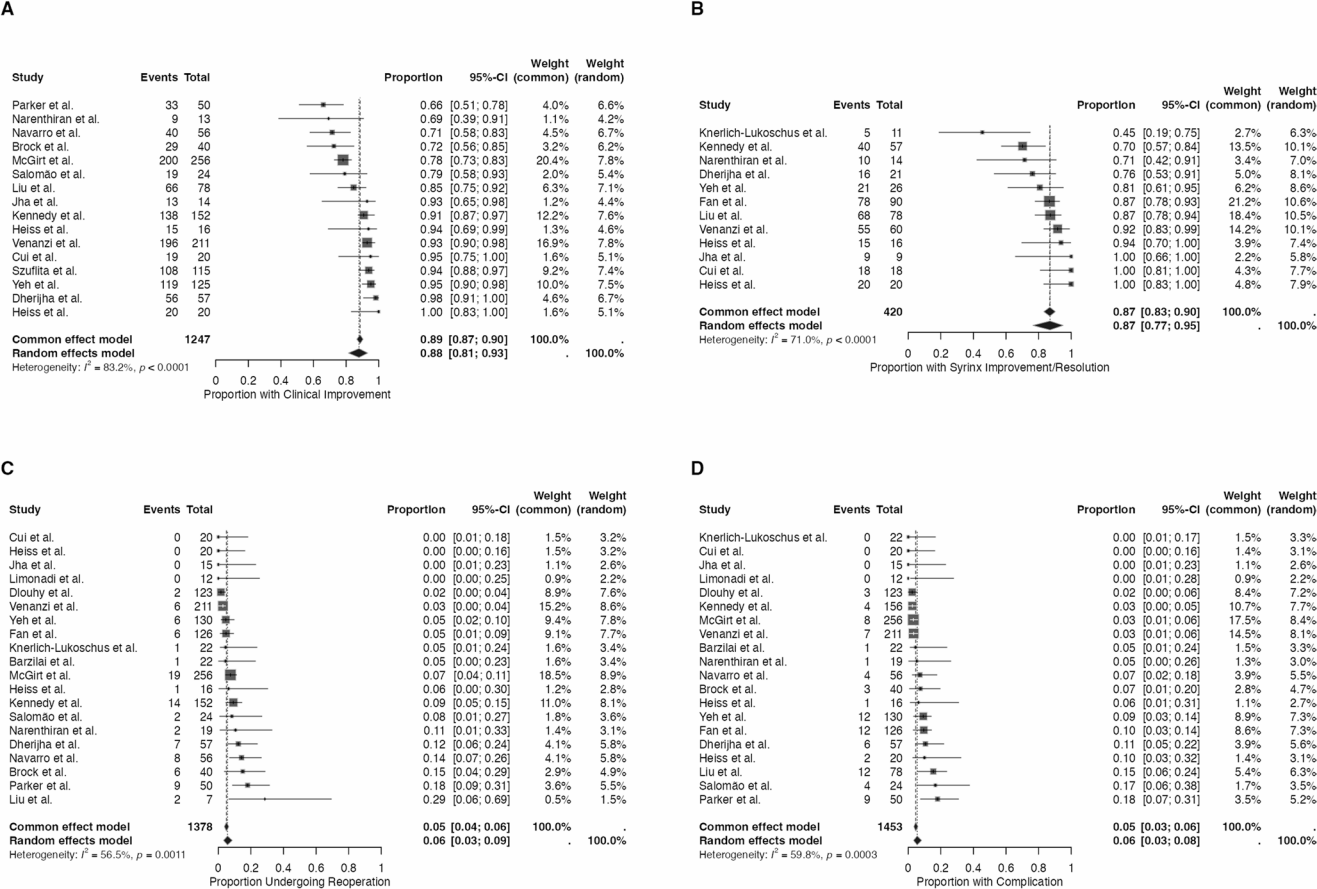
Forest plots showing pooled proportions of (**A**) clinical improvement, (**B**) syrinx improvement/resolution, (**C**) reoperation, and (**D**) complications following IOUS-guided surgery in CM-I using a random-effects model

Sixteen studies were included in the meta-analysis of the clinical improvement rate (Fig. 2A). The meta-analysis reported a pooled clinical improvement rate of 88% (95% CI: 81%−93%). The meta-regression indicated that a higher mean disease duration (Estimate = 0.474, *P* = 0.004) was associated with higher clinical improvement rates (Table 3). Twelve studies were included in the meta-analysis of syrinx improvement/resolution rate (Fig. 2B). The meta-analysis showed a pooled syrinx improvement/resolution rate of 87% (95% CI: 77%−95%). The meta-regression demonstrated that a longer mean disease duration (Estimate = 0.558, *P* = 0.043), presence of motor deficit (Estimate = 0.048, *P* = 0.003), presence of sensory deficit (Estimate = 0.044, *P* < 0.001) and duraplasty (Estimate = 0.016, *P* = 0.005) were associated with more favorable syrinx improvement/resolution rates. In contrast, bone-only decompression (Estimate = −0.013, *P* = 0.002) was linked to lower syrinx improvement/resolution rates (Table 3).

**Table 3:** Meta-Regression results for IOUS in CM-I

Outcome	Modifier	Coefficient	SE	Z	*P*-value	Tau2
Clinical Improvement	Mean Age	−0.002	0.017	−0.148	0.883	0.712
Clinical Improvement	Mean Disease Duration	0.474	0.164	2.895	0.004	0.082
Clinical Improvement	Male (%)	−0.006	0.020	−0.284	0.777	0.595
Clinical Improvement	Headache (%)	−0.011	0.013	−0.820	0.412	0.478
Clinical Improvement	Motor Deficit (%)	−0.015	0.014	−1.117	0.264	0.317
Clinical Improvement	Sensory Deficit (%)	0.000	0.021	−0.005	0.996	0.705
Clinical Improvement	Cerebellar Signs (%)	0.006	0.035	0.179	0.858	0.610
Clinical Improvement	Cranial Nerve Symptoms (%)	−0.033	0.048	−0.697	0.486	0.611
Clinical Improvement	Preoperative Syrinx (%)	0.007	0.008	0.833	0.405	0.665
Clinical Improvement	Preoperative Hydrocephalus (%)	−0.025	0.048	−0.532	0.595	0.775
Clinical Improvement	Bone-Only Decompression (%)	0.005	0.007	0.736	0.462	0.641
Clinical Improvement	Duraplasty (%)	0.004	0.007	0.503	0.615	0.703
Syrinx Improvement/Resolution	Mean Age	0.022	0.016	1.313	0.189	0.410
Syrinx Improvement/Resolution	Mean Disease Duration	0.558	0.276	2.024	0.043	0.000
Syrinx Improvement/Resolution	Male (%)	−0.002	0.022	−0.072	0.943	0.576
Syrinx Improvement/Resolution	Headache (%)	0.009	0.018	0.484	0.629	0.582
Syrinx Improvement/Resolution	Motor Deficit (%)	0.048	0.016	2.958	0.003	0.017
Syrinx Improvement/Resolution	Sensory Deficit (%)	0.044	0.012	3.699	0.000	0.019
Syrinx Improvement/Resolution	Cerebellar Signs (%)	0.054	0.030	1.774	0.076	0.429
Syrinx Improvement/Resolution	Cranial Nerve Symptoms (%)	0.015	0.052	0.292	0.770	0.600
Syrinx Improvement/Resolution	Preoperative Syrinx (%)	0.013	0.010	1.314	0.189	0.442
Syrinx Improvement/Resolution	Preoperative Hydrocephalus (%)	0.049	0.040	1.210	0.226	0.332
Syrinx Improvement/Resolution	Bone-Only Decompression (%)	−0.013	0.004	−3.125	0.002	0.088
Syrinx Improvement/Resolution	Duraplasty (%)	0.016	0.006	2.828	0.005	0.202
Reoperation	Mean Age	0.022	0.011	2.116	0.034	0.180
Reoperation	Mean Disease Duration	−0.055	0.172	−0.321	0.748	0.054
Reoperation	Male (%)	−0.018	0.015	−1.251	0.211	0.232
Reoperation	Headache (%)	0.011	0.010	1.180	0.238	0.209
Reoperation	Motor Deficit (%)	0.013	0.012	1.055	0.291	0.140
Reoperation	Sensory Deficit (%)	−0.005	0.014	−0.355	0.723	0.156
Reoperation	Cerebellar Signs (%)	−0.007	0.022	−0.295	0.768	0.320
Reoperation	Cranial Nerve Symptoms (%)	0.019	0.026	0.758	0.449	0.135
Reoperation	Preoperative Syrinx (%)	−0.003	0.008	−0.407	0.684	0.311
Reoperation	Preoperative Hydrocephalus (%)	0.028	0.040	0.697	0.486	0.396
Reoperation	Bone-Only Decompression (%)	0.003	0.005	0.521	0.602	0.325
Reoperation	Duraplasty (%)	−0.005	0.005	−0.870	0.384	0.316
Complication	Mean Age	0.024	0.011	2.177	0.029	0.241
Complication	Mean Disease Duration	0.100	0.225	0.447	0.655	0.262
Complication	Male (%)	−0.013	0.018	−0.703	0.482	0.471
Complication	Headache (%)	0.014	0.011	1.190	0.234	0.345
Complication	Motor Deficit (%)	0.025	0.014	1.820	0.069	0.158
Complication	Sensory Deficit (%)	0.011	0.016	0.689	0.491	0.238
Complication	Cerebellar Signs (%)	−0.024	0.021	−1.143	0.253	0.234
Complication	Cranial Nerve Symptoms (%)	−0.028	0.036	−0.786	0.432	0.422
Complication	Preoperative Syrinx (%)	0.006	0.007	0.858	0.391	0.326
Complication	Preoperative Hydrocephalus (%)	0.035	0.036	0.973	0.331	0.441
Complication	Bone-Only Decompression (%)	−0.003	0.005	−0.530	0.596	0.355
Complication	Duraplasty (%)	0.004	0.005	0.850	0.396	0.363

Twenty studies were included in the meta-analysis of reoperation rate (Fig. 2C). The meta-analysis found a pooled reoperation rate of 6% (95% CI: 3%−9%). The meta-regression indicated that older age (Estimate = 0.022, *P* = 0.034) was linked to higher reoperation rates (Table 3). Twenty studies were included in the meta-analysis of complication rate (Fig. 2D). The meta-analysis showed a pooled complication rate of 6% (95% CI: 3%−8%). The meta-regression indicated that older age (Estimate = 0.024, *P* = 0.034) was linked to higher complication rates (Table 3).

### Sensitivity analysis

The sensitivity analysis for the clinical improvement rates showed that the results were robust, and the pooled estimates remained consistent (Supplementary Fig. S1A). The sensitivity analysis for the syrinx improvement/resolution showed that none of the included studies had a significant impact on the pooled estimates, and the results remained consistent (Supplementary Fig. S1B). The sensitivity analysis for the reoperation and complication rate showed minor fluctuations in the pooled estimates; however, the results were robust, and none of the included studies had a significant impact on the results (Supplementary Fig. S1C-D).

### Publication bias

The funnel plot for the clinical improvement rate showed a symmetrical pattern, and Egger’s test (*P* = 0.79) indicated no evidence of publication bias (Supplementary Fig. S2A). The funnel plot for the syrinx improvement/resolution appeared symmetrical, and Egger’s test (*P* = 0.97) showed no significant publication bias (Supplementary Fig. S2B). The funnel plot for the reoperation rate was fairly symmetrical, and Egger’s test (*P* = 0.37) showed no evidence of publication bias (Supplementary Fig. S2C). The funnel plot for complication rates showed mild asymmetry; however, Egger’s test (*P* = 0.26) showed no significant publication bias (Supplementary Fig. S2D).

### GRADE assessment

The GRADE assessment rated the certainty of evidence as low across all evaluated endpoints, including clinical improvement, syrinx resolution, reoperation, and complication rates (Supplementary Table S6). This downgrading mainly reflects the non-randomized design of the studies, moderate-to-serious risk of bias, and diversity in outcome definitions and surgical approaches. Despite these limitations, the pooled estimates were consistently promising, with favorable rates of clinical and radiological improvement and low rates of reoperation and complications.

## Discussion

This systematic review and meta-analysis highlight the increasing utilization of IOUS as a guiding tool in the surgical management of CM-I. Our pooled analysis of 1,576 patients revealed a high rate of clinical improvement (88%) and syrinx resolution or improvement (87%), accompanied by low rates of reoperation (6%) and complications (6%). These findings suggest that IOUS can help surgeons make more informed surgical decisions and potentially improve clinical and radiological outcomes while reducing morbidity.

Historically, the extent of PFD, particularly the decision to open the dura, has been controversial [12, 45–47]. While duraplasty may enhance cerebrospinal fluid (CSF) dynamics and improve syrinx-related outcomes, it is associated with a higher risk of pseudomeningocele, CSF leak, and infection [12, 45–47]. In a meta-analysis of 13 studies by Lin et al., they demonstrated that PFD with duraplasty was associated with an increase in operative time compared to PFD without duraplasty, a higher risk of clinical improvement in syringomyelia, yet a higher risk of CSF-related adverse events, pseudomeningocele, and a lower risk of recurrence [46]. Tam et al., in a meta-analysis of 17 studies, demonstrated that PFD with duraplasty can achieve more favorable clinical outcomes in cases without hydrocephalus and craniocervical region instability; however, it is associated with a higher risk of complications [12]. Our meta-regression indicated that duraplasty was independently associated with better syrinx outcomes, supporting the idea that incorporating IOUS into an individualized surgical strategy may achieve an optimal balance between efficacy and safety.

Several studies have evaluated the role of IOUS in CM-I surgery [15–35]. For instance, Yeh et al. indicated that the application of IOUS enables surgeons to avoid duraplasty in 40 of 130 children when sufficient decompression and CSF flow restoration are seen intraoperatively, leading to no adverse events in the bone-only group compared to 12 complications in the duraplasty group [32]. Dherijha et al. used IOUS and achieved favorable outcomes in 98% of cases, with dural opening performed in only 6% of cases and a 12% reoperation rate [17].

Intraoperative stratification of decompression extent is also another advantage of IOUS application through assessment of CSF flow dynamics and tonsillar pulsatility. Brock et al., in a prospective analysis of 49 cases, demonstrated that CSF velocity > 3 cm/s following bony decompression had promising results without duraplasty [23]. In contrast, those with impaired flow had dural opening and were also associated with promising results and lower complication rates in the non-duraplasty cases [23]. In a study by Szuflita et al., they showed that IOUS application can help surgeons identify cases that may benefit from further procedures like fourth ventricle stenting when CSF flow remains obstructed after bony decompression [18].

Age can significantly influence the management of CM-I cases. Dlouhy et al. compared 78 children with 45 adults with CM-I who were treated using IOUS [22]. They reported a 0% complication rate in the children’s group receiving extradural and intradural decompression with autologous cervical fascia duraplasty compared to 6.7% in the adult group, such as aseptic meningitis and CSF leak [22]. These findings were aligned with our meta-regression that indicated that older age was considerably associated with higher rates of complications (*P* = 0.034) and reoperations (*P* = 0.034). These findings indicate that the pediatric population can benefit from a more robust postoperative recovery and a reduced likelihood of complications. The advantage of IOUS to personalize decompression intraoperatively may be especially helpful in adult patients, where the margin for surgical safety is narrower.

Pediatric CM represents an evolving and dynamic condition, often associated with ongoing cranial and spinal growth that can influence postoperative outcomes over time [5, 11, 48, 49]. In children, bone-only decompression is frequently favored when IOUS demonstrates adequate CSF flow restoration and tonsillar pulsatility, thereby allowing for the avoidance of dural opening and reducing the risk of postoperative CSF-related complications [11, 26, 27, 32]. In contrast, in adults, duraplasty is more commonly performed to minimize the risk of symptom recurrence and syrinx persistence [11, 28, 30, 50]. The International Consensus on Chiari Malformation and Syringomyelia specifically cautions that bone-only decompression in symptomatic CM-I without syringomyelia is generally not recommended due to recurrence risk (agreement 75%, Grade D) [11]. This distinction highlights the importance of age-tailored surgical strategies and underscores the role of IOUS in guiding the extent of decompression across pediatric and adult populations.

While our findings suggested that IOUS-guided surgery for CM-I cases can lead to considerable syrinx improvement rates, syrinx persistence or progression continues to be an issue in a group of cases. Our pooled syrinx resolution/improvement rate of 87% is consistent with the study of Liu et al., which showed comparable syrinx results with and without craniectomy, incorporating IOUS to guide tonsillar resection and cisterna magna reconstruction [19]. Szuflita et al. indicated that in cases with tonsillar enlargement following surgery, in the absence of clinical presentations, these cases can be managed non-operatively [18]. This indicates that radiological evidence by itself might not necessitate reoperation. Although IOUS may assist in optimizing decompression and contribute to favorable syrinx outcomes, its utility is context-dependent. In cases of extensive or holocord syringomyelia, dural decompression is generally indicated regardless of IOUS findings. In contrast, IOUS is particularly valuable in small or localized cervical syringomyelia to confirm adequate CSF flow restoration and potentially avoid unnecessary dural opening [12, 46, 47].

The findings of the current study highlight the application of IOUS, a non-invasive tool that guides intraoperative decision-making, minimizing surgical complications while maintaining clinical efficacy. Utilization of the IOUS facilitates intraoperative assessment of CSF flow dynamics, tonsillar pulsatility, and sufficiency of decompression. The application of IOUS can help surgeons decide whether to perform duraplasty and may help avoid unnecessary intradural manipulation. IOUS is especially helpful in complex cases, as it provides immediate feedback and enhances surgical precision. However, the application of IOUS requires expertise and experience, as image interpretation heavily depends on the operator and can be influenced by various technical variables such as probe positioning and artifacts from bone or dural reflection. Additionally, the lack of standardized protocols and quantitative thresholds for determining sufficient decompression limits the generalizability of the IOUS application in different institutions. While IOUS provides individualized decompression strategies, future prospective studies are required to define reproducible imaging outcomes and incorporate IOUS findings with long-term results.

## Limitations

Our study has some limitations that need to be acknowledged. The majority of the included studies were retrospective, which can introduce confounding bias. Another limitation is that, despite conducting a robust meta-analytical approach, all outcomes were rated as having a low level of certainty in the GRADE assessment. This suggests that the actual effect may differ substantially from our estimates, primarily due to the non-randomized nature of the studies and moderate-to-serious RoB, particularly in the confounding domain. Another limitation was the high heterogeneity in the endpoint, which stems from diversity in surgical approaches, the application and interpretation of IOUS, and outcome definitions, potentially influencing the generalizability of the results. To overcome this issue, we conducted a meta-regression to identify the sources of heterogeneity. Finally, long-term results were not consistently reported, which limits our ability to assess the durability of IOUS-guided surgical benefits. Additionally, the follow-up periods varied significantly across the included studies, and many did not offer long-term follow-up data. This variation restricts the ability to accurately assess the durability of IOUS-guided surgery outcomes, especially concerning syrinx recurrence and the actual need for reoperation, which may be underestimated in shorter follow-up times.

All included studies mainly involved CM-I and cases of Chiari 1.5, which generally require dural decompression due to brainstem descent. These were rarely reported and not analyzed separately. Therefore, the findings mainly reflect the use of IOUS in Chiari I, with limited applicability to Chiari 1.5. Reporting of radiologic endpoints across the included studies was heterogeneous. Syrinx extension was measured inconsistently, with several studies providing only qualitative descriptions, and definitions of postoperative improvement ranged from quantitative thresholds to subjective assessments. Moreover, only a small subset of studies measured postoperative tonsillar position, limiting our ability to harmonize or compare tonsillar ascent across the evidence base. This variability may introduce residual heterogeneity in our pooled radiologic outcomes. Complication reporting was heterogeneous, and most studies did not distinguish postoperative adverse events between bone-only decompression and duraplasty. As a result, the available data were insufficient for a reliable stratified complication analysis. This limitation likely underestimates the true difference in CSF-related complication profiles between the two approaches, which has been well-documented in prior literature. Operative details were variably reported, and none of the included studies explicitly mentioned using tonsillar coagulation. However, because older studies sometimes provided limited intraoperative descriptions, the possibility of unreported technique variation, including rare use of outdated tonsillar reduction methods, cannot be fully excluded and may contribute to residual heterogeneity. Standardized clinical outcome scales were not used across the included studies; none employed the Chicago Chiari Outcome Scale, and most relied on non-uniform, study-specific definitions of clinical improvement, contributing to substantial heterogeneity in outcome reporting.

Future studies should focus on prospective, multicenter research with standardized protocols for IOUS application in CM-I surgery. By standardizing IOUS metrics such as CSF flow dynamics or tonsillar pulsatility thresholds, outcomes and decision-making processes can be improved. Long-term follow-up is also necessary to evaluate the durability of outcomes related to IOUS-guided surgery.

## Conclusion

IOUS is a valuable adjunct in the surgical management of CM-I patients and provides real-time intraoperative guidance that can optimize decompression while reducing complications. Our findings indicate that IOUS-guided surgery is associated with high rates of clinical improvement and syrinx resolution, along with low reoperation and complication rates. IOUS may assist surgeons in customizing surgical approaches by guiding intraoperative decisions on whether to open the dura, which can prevent unnecessary intradural manipulation and help reduce complication rates. Further large, prospective, multi-center studies with standardization of the IOUS protocols and outcome definitions are required to confirm the findings of our study and enhance the generalizability and clinical adoption of IOUS-guided surgery in CM-I patients.

## Supplementary Information

Below is the link to the electronic supplementary material. 

Supplementary material 1 (DOCX 773 KB) 

## Data Availability

“The data supporting this study’s findings are available from the corresponding author upon reasonable request.”

## Declarations

### Clinical trial number

“Not applicable.”

### Grammarly usage

“Grammarly was used to enhance the clarity and readability of the manuscript.”“This manuscript complies with all instructions to authors. All authors meet authorship criteria and have approved the final manuscript. This manuscript has not been published and is not under consideration elsewhere. A PRISMA checklist was used and is included with the submission.”
