# Supplementary material for: Intraoperative ultrasonography in the surgical management of Chiari I malformation: A systematic review and meta-analysis of outcomes and applications
Source: Neurol Sci. 2026 Mar 9;47(4):328. doi: 10.1007/s10072-026-08879-8 (PMC12968098; doi:10.1007/s10072-026-08879-8)
Supplement: Supplementary file 1 — Supplementary material 1 (DOCX 773 KB) [file 10072_2026_8879_MOESM1_ESM.docx]

Supplementary File

Intraoperative Ultrasonography in the Surgical Management of Chiari I Malformation: A Systematic Review and Meta-Analysis of Outcomes and Applications

| Supplementary Table S1. Search strategy | |
| --- | --- |
| PubMed | ("Arnold-Chiari Malformation"[Mesh] OR "chiari malformation type 1"[tiab] OR "chiari malformation type I"[tiab] OR "chiari 1 malformation"[tiab] OR "chiari malformation type-1"[tiab] OR "chiari malformation type-I"[tiab] OR CM-1[tiab] OR "CM 1"[tiab]) AND ("Intraoperative Period"[Mesh] OR intraoperat*[tiab] OR intraop*[tiab]) AND ("Ultrasonography"[Mesh] OR ultrasound*[tiab] OR ultrasonograph*[tiab] OR sonograph*[tiab]) |
| Embase | ('chiari malformation'/exp OR 'chiari malformation type i':ti,ab OR 'chiari type i':ti,ab OR 'chiari 1 malformation':ti,ab OR 'chiari malformation type-1':ti,ab OR 'chiari malformation type-I':ti,ab OR 'CM 1':ti,ab OR 'CM-1':ti,ab) AND ('intraoperative period'/exp OR intraoperat*:ti,ab OR intraop*:ti,ab) AND ('ultrasonography'/exp OR ultrasound*:ti,ab OR ultrasonograph*:ti,ab OR sonograph*:ti,ab) |
| Scopus | TITLE-ABS-KEY(("chiari malformation" OR "chiari malformation type 1" OR "chiari malformation type I" OR "chiari 1 malformation" OR "chiari malformation type-1" OR "chiari malformation type-I" OR "arnold-chiari malformation" OR "arnold chiari malformation" OR "CM-1" OR "CM 1")) AND TITLE-ABS-KEY(("intraoperative" OR "intraoperative period" OR intraoperat* OR intraop*)) AND TITLE-ABS-KEY(("ultrasound" OR "ultrasonography" OR "sonography" OR "ultrasonograph*" OR "sonograph*" OR "intraoperative ultrasound")) |
| Web of Science | TS=("chiari malformation" OR "chiari malformation type 1" OR "chiari malformation type I" OR "chiari 1 malformation" OR "chiari malformation type-1" OR "chiari malformation type-I" OR "arnold-chiari malformation" OR "arnold chiari malformation" OR "CM-1" OR "CM 1") AND TS=("intraoperative" OR "intraoperative period" OR intraoperat* OR intraop*)  AND TS=("ultrasound" OR "ultrasonography" OR "sonography" OR "ultrasonograph*" OR "sonograph*" OR "intraoperative ultrasound") |
| Search date = July 5, 2025 | |

| Supplementary Table S2. PICO framework | |
| --- | --- |
| Population (P) | Patients with Chiari I Malformation undergoing surgical treatment. |
| Intervention (I) | Use of Intraoperative Ultrasonography (IOUS) during posterior fossa decompression surgery (with or without duraplasty). |
| Comparison (C) | Surgical treatment without IOUS |
| Outcome (O) | Clinical improvement, Syrinx resolution, Reoperation rates, Surgical complications |

| Supplementary Table S3. Extracted variables | |
| --- | --- |
| Baseline | Outcome |
| Digital Object Identifier  First Author and Study Title  Year of Publication  Study Design  Country of Study  Participating Institution(s)  Number of Patients  Mean Age of Patients  Number of Male Patients  Number of Female Patients  Mean Duration of Symptoms Before Surgery  Presence of Preoperative Headache Symptoms  Presence of Preoperative Motor Deficits  Presence of Preoperative Sensory Disturbances  Presence of Preoperative Cerebellar Signs  Presence of Preoperative Cranial Nerve Deficits  Number of Patients with Preoperative Syringomyelia  Number of Patients with Preoperative Hydrocephalus  Number of Patients Undergoing Intraoperative Ultrasonography (IOUS)  Number of Patients Undergoing Bone-Only Decompression  Number of Patients Undergoing Decompression with Duraplasty  Number of Patients Undergoing Dural Opening | Mean Duration of Follow-Up  Number of Patients with Clinical Improvement Postoperatively  Total Number of Patients Assessed for Clinical Improvement  Number of Patients with Syrinx Resolution or Improvement  Total Number of Patients with Preoperative Syrinx  Number of Patients Undergoing Reoperation  Total Number of Patients at Risk for Reoperation  Number of Patients Experiencing Postoperative Complications  Total Number of Patients at Risk for Complications |
|  |  |
|  |  |

| Supplementary Table S4. Definition of Primary and Secondary Outcomes | |
| --- | --- |
| Outcome | Definition Used in This Review |
| Primary Outcome | |
| Clinical improvement | Postoperative improvement or resolution of preoperative symptoms (eg, headache, sensory or motor deficits, cerebellar dysfunction), as defined by each individual study. |
| Secondary Outcomes | |
| Syrinx resolution | Complete disappearance of the syringomyelia cavity on postoperative MRI. |
| Syrinx improvement | Partial reduction in syrinx size on postoperative imaging. |
| Syrinx improvement/ resolution (combined) | Studies reporting both categories were pooled into a combined endpoint for meta-analysis. |
| Reoperation | Any subsequent surgical intervention after the initial decompression (for persistent symptoms, syrinx progression, CSF leak, pseudomeningocele, or inadequate decompression). |
| Complications | Any postoperative adverse event reported in the study, including CSF leak, pseudomeningocele, aseptic meningitis, wound infection, or neurological worsening. |

| Supplementary Table S5. Risk of Bias assessment with MINORS tool | | | | | | | | | | | | | |
| --- | --- | --- | --- | --- | --- | --- | --- | --- | --- | --- | --- | --- | --- |
| Study | Item 1 | Item 2 | Item 3 | Item 4 | Item 5 | Item 6 | Item 7 | Item 8 | Item 9 | Item 10 | Item 11 | Item 12 | Overall |
| Venanzi et al., 2024 | 2 | 2 | 2 | 0 | 2 | 2 | 0 | 2 | 2 | 2 | 0 | 2 | 18/24 |
| Jha et al., 2024 | 2 | 2 | 2 | 2 | 2 | 2 | 0 | 0 | 2 | 2 | 2 | 2 | 20/24 |
| Dherijha et al., 2024 | 2 | 2 | 2 | 2 | 2 | 2 | 2 | 0 | 0 | 2 | 2 | 2 | 20/24 |
| Szuflita et al., 2021 | 2 | 2 | 2 | 2 | 2 | 2 | 0 | 0 | 0 | 2 | 2 | 2 | 18/24 |
| Liu et al., 2020 | 2 | 2 | 2 | 2 | 2 | 2 | 2 | 0 | 2 | 2 | 2 | 2 | 22/24 |
| Salomão et al., 2019 | 2 | 2 | 2 | 2 | 2 | 2 | 2 | 0 | 0 | 0 | 0 | 2 | 14/24 |
| Knerlich-Lukoschus et al., 2019 | 2 | 2 | 2 | 2 | 2 | 2 | 2 | 0 | 0 | 0 | 0 | 2 | 14/24 |
| Dlouhy et al., 2018 | 2 | 2 | 2 | 2 | 2 | 2 | 2 | 0 | 0 | 0 | 0 | 2 | 14/24 |
| Brock et al., 2017 | 2 | 2 | 2 | 2 | 2 | 2 | 0 | 0 | 2 | 2 | 2 | 2 | 20/24 |
| Fan et al., 2017 | 2 | 2 | 2 | 2 | 2 | 2 | 2 | 0 | 2 | 2 | 2 | 2 | 22/24 |
| Barzilai et al., 2016 | 2 | 2 | 2 | 2 | 2 | 2 | 2 | 0 | 0 | 0 | 0 | 2 | 16/24 |
| Kennedy et al., 2015 | 2 | 2 | 2 | 2 | 2 | 2 | 2 | 0 | 0 | 0 | 0 | 2 | 16/24 |
| Narenthiran et al., 2015 | 2 | 2 | 2 | 2 | 2 | 2 | 2 | 0 | 0 | 0 | 0 | 2 | 16/24 |
| Parker et al., 2013 | 2 | 2 | 2 | 2 | 2 | 2 | 0 | 0 | 2 | 2 | 2 | 2 | 20/24 |
| Cui et al., 2011 | 2 | 2 | 2 | 2 | 2 | 2 | 2 | 0 | 0 | 0 | 0 | 2 | 16/24 |
| Heiss et al., 2010 | 2 | 2 | 2 | 2 | 2 | 2 | 2 | 0 | 0 | 0 | 0 | 2 | 16/24 |
| McGirt et al., 2008 | 2 | 2 | 2 | 2 | 2 | 2 | 0 | 0 | 2 | 2 | 2 | 2 | 20/24 |
| Yeh et al., 2006 | 2 | 2 | 2 | 2 | 2 | 2 | 2 | 0 | 0 | 0 | 0 | 2 | 16/24 |
| Limonadi et al., 2004 | 2 | 2 | 2 | 2 | 2 | 2 | 2 | 0 | 0 | 0 | 0 | 2 | 16/24 |
| Navarro et al., 2004 | 2 | 2 | 2 | 2 | 2 | 2 | 0 | 0 | 0 | 0 | 0 | 2 | 14/24 |

**
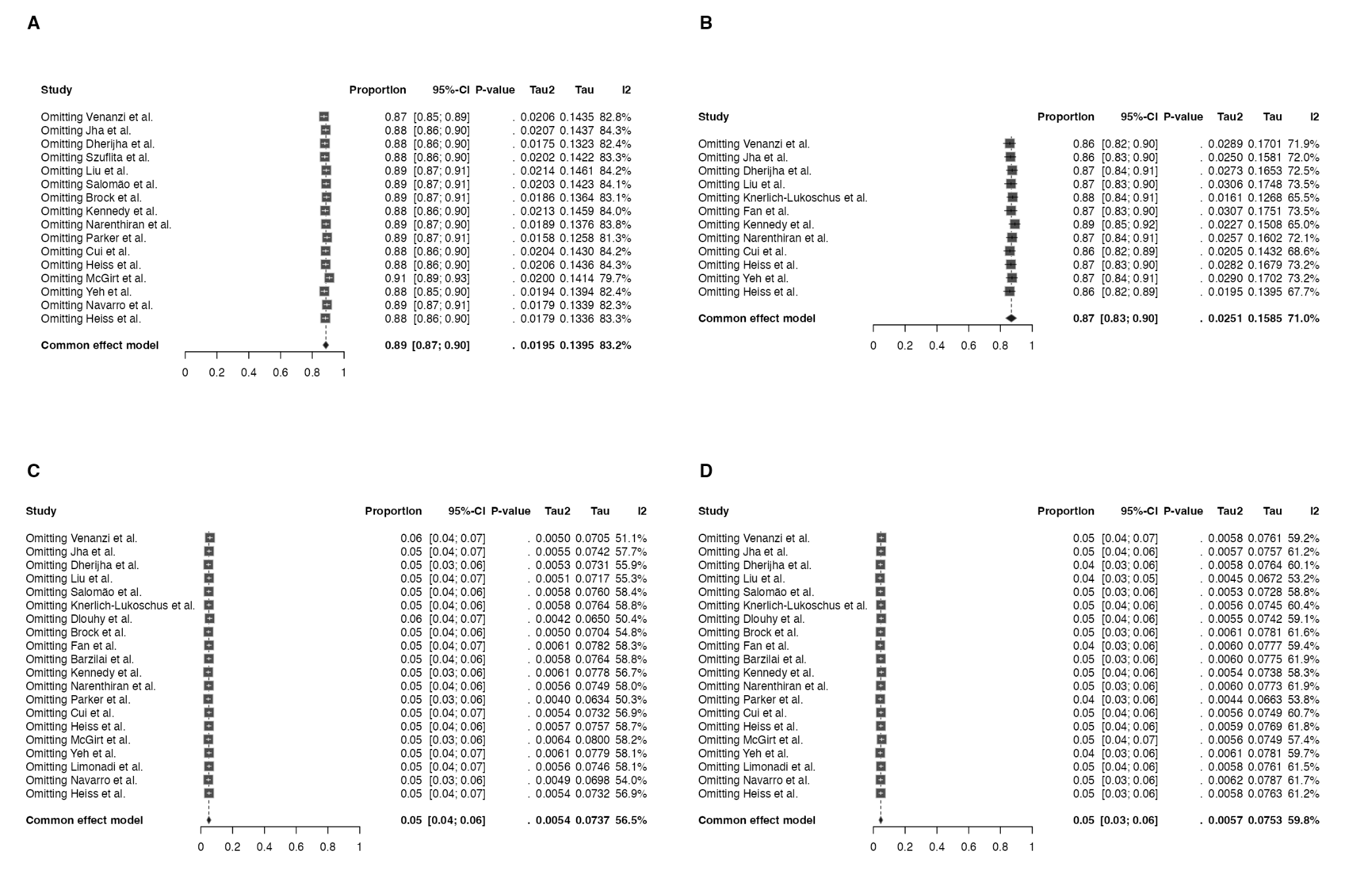
**

**Supplementary Figure S1**. Leave-One-Out Sensitivity Analyses for Outcomes (A) Clinical improvement, (B) syrinx improvement/resolution, (C) reoperation rate, and (D) complication rate following IOUS in the surgical management of CM-I.


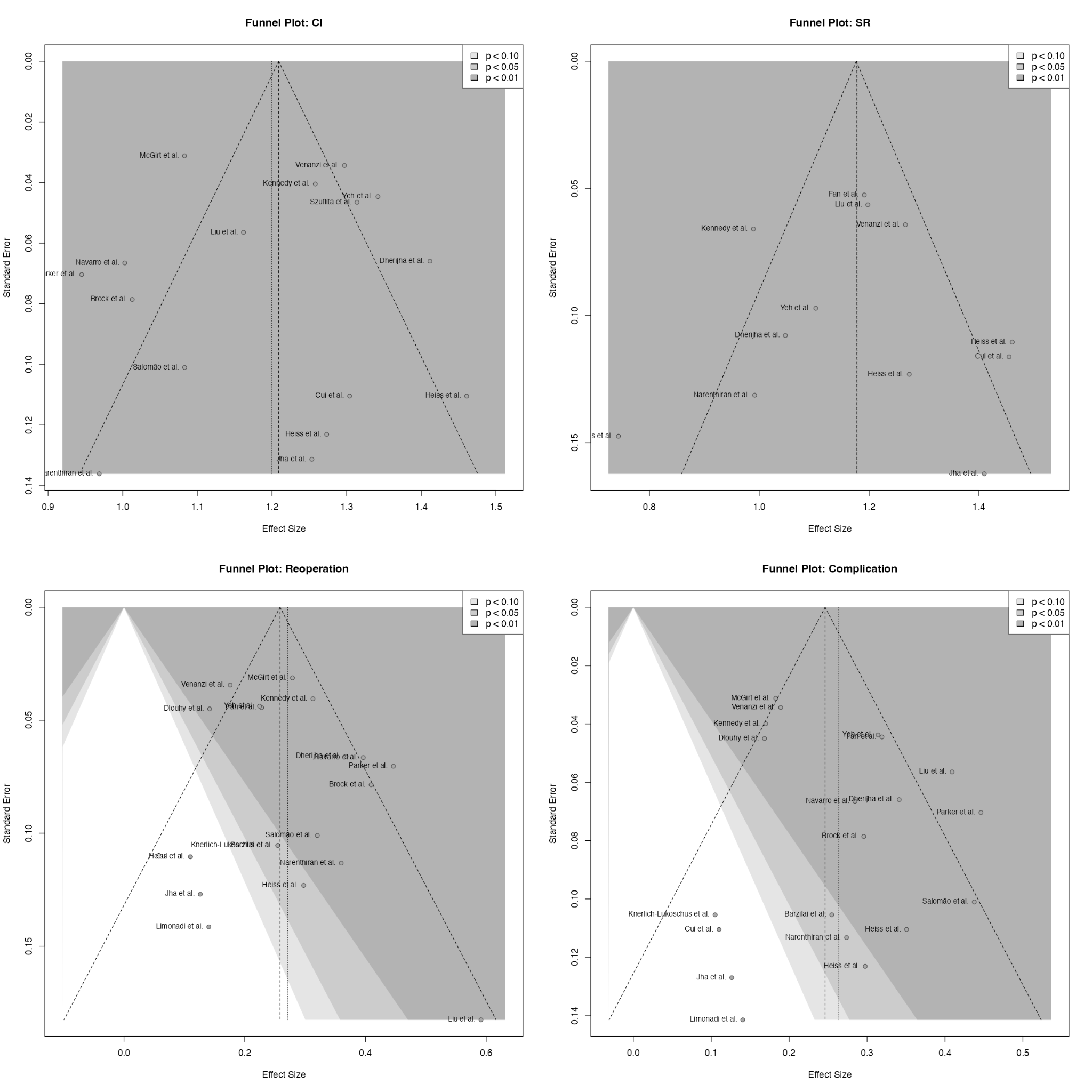


**Supplementary Figure S2**. Funnel plots evaluating publication bias across outcomes for (A) clinical improvement, (B) syrinx improvement/resolution, (C) reoperation rate, and (D) complication rate following IOUS in the surgical management of CM-I.

| Supplementary Table S6. GRADE Assessment Summary | | |
| --- | --- | --- |
| Outcome | Certainity | Reasons for Downgrading |
| Clinical Improvement | Low | Non-randomized studies; moderate-to-serious risk of bias (confounding); heterogeneity |
| Syrinx Improvement/Resolution | Low | Non-randomized studies; moderate RoB; outcome subject to imaging variability; some inconsistency |
| Reoperation Rate | Low | Observational data; moderate-to-serious RoB; varying surgical techniques and definitions |
| Complication Rate | Low | Non-randomized studies; moderate RoB; inconsistency in complication reporting |
